# Draft Genome Sequences of Three Type Strains, Acetobacter farinalis KACC 21251, Acetobacter suratthaniensis KACC 21252, and Acetobacter thailandicus KACC 21253

**DOI:** 10.1128/mra.00246-23

**Published:** 2023-05-09

**Authors:** Seunghwan Kim, Hyorim Choi, Yiseul Kim, Daseul Lee, Byeong-Hak Han, Seung-Beom Hong, Soon-Wo Kwon, Jun Heo

**Affiliations:** a Agricultural Microbiology Division, National Institute of Agricultural Sciences, Rural Development Administration, Wanju-gun, Jeollabuk-do, Republic of Korea; b Division of Biotechnology, Chonbuk National University, Iksan, Republic of Korea; University of Southern California

## Abstract

As part of a genome database construction of type strains, we report the draft genome sequences of three strains of acetic acid bacteria, i.e., Acetobacter farinalis KACC 21251^T^, Acetobacter suratthaniensis KACC 21252^T^, and Acetobacter thailandicus KACC 21253^T^.

## ANNOUNCEMENT

The genus *Acetobacter* is a genus of acetic acid bacteria that convert ethanol to acetic acid in the presence of oxygen (1, 2). Three *Acetobacter* type strains, i.e., Acetobacter farinalis strain KACC 21251 (3), Acetobacter suratthaniensis strain KACC 21252 (4), and Acetobacter thailandicus strain KACC 21253 (5), were isolated from fermented starch(khao-khab), fruit, and flower (blue trumpet vine) respectively, and were deposited in the Korean Agricultural Culture Collection (KACC). As part of a genome database construction project involving strains deposited in the KACC, genomic analyses were conducted. The bacteria were grown in Reasoner's 2A (R2A) broth from a single colony for 24 h at 28°C with agitation. Genomic DNA was extracted from the cultured cells using the Qiagen MagAttract high-molecular-weight (HMW) DNA kit (Qiagen, Hilden, Germany). The concentration of the extracted DNA was quantified using a Qubit 2.0 fluorometer (Invitrogen, Carlsbad, CA, USA). Contamination of DNA or cultured strains was checked by sequencing the 16S rRNA gene (6) using the ABI 3730 DNA sequencing system (Applied Biosystems, Foster City, CA, USA). In order to generate three different libraries, genomic DNA was fragmented to approximately 550 bp using the M220 focused ultrasonicator (Covaris Ltd., Brighton, UK), fragmented DNA was quantified using the 2100 Bioanalyzer (Agilent, Palo Alto, CA, USA) with the DNA 7500 kit, and then the libraries were constructed using the TruSeq DNA low-throughput (LT) library preparation kit (Illumina, San Diego, CA, USA). Three different whole-genome sequencing processes were performed on the Illumina MiSeq system with 2 × 300-bp paired-end reads using the 600-cycle sequencing kit (MiSeq reagent kit v3). The quality of the sequencing data was then controlled with Trimmomatic v0.36 with default options, and PhiX sequences were removed by BBmap v38.22 with default options. The polished reads were assembled with SPAdes v3.13.0 with default options using 16 threads (7). Genome and sequencing parameters are shown in Table 1. The assembled genomes were annotated by the NCBI Prokaryotic Genome Annotation Pipeline (PGAP) (8) using the best-placed reference protein set (GeneMarkS-2+). The annotation results for the three genomes are also listed in Table 1. In addition, the three strains were annotated in terms of carbohydrate metabolism- and carbon metabolism-related enzymes by dbCAN2 with default parameters (9) with the hidden Markov model (HMM) database search tool v10 (10) in the KBase environment (11). The results showed that genes for glycoside hydrolases, glycosyltransferases, carbohydrate esterases, polysaccharide lyases, and auxiliary activities were found in the three genomes.

**TABLE 1:** Sequencing, assembly, and annotation results for the sequenced strains

Parameter	Data for strain:		
	KACC 21251	KACC 21252	KACC 21253
BioSample accession no.	SAMN31703510	SAMN31703545	SAMN31703546
BioProject accession no.	PRJNA901062	PRJNA901062	PRJNA901062
GenBank accession no.	JAPIUX000000000	JAPIUY000000000	JAPIUZ000000000
SRA accession no.	SRX18280854	SRX18280856	SRX18280855
Total no. of reads	3,028,852	3,145,728	3,209,840
Sequencing depth (×)	227.83	207.54	266.31
No. of contigs	71	50	28
Contig *N*_50_ (bp)	192,191	226,626	431,044
Genome size (bp)	2,952,797	3,108,004	2,805,005
G+C content (%)	59.1	59.0	50.5
Total no. of coding sequences	2,614	2,836	2,534
No. of rRNA operons	1	1	1
No. of tRNAs	47	45	49
No. of noncoding RNAs	4	4	4
No. of CRISPR arrays	1	0	0

### Data availability.

The genomes are available in GenBank under the accession numbers shown in Table 1.

## References

[1] Kersters K, Lisdiyanti P, Komagata K, Swings J. 2006. The family Acetobacteraceae: the genera *Acetobacter*, *Acidomonas*, *Asaia*, *Gluconacetobacter*, *Gluconobacter*, and *Kozakia*, p 163–200. *In* Dworkin M, Falkow S, Rosenberg E, Schleifer K-H, Stackebrandt E (ed), The prokaryotes, vol 5. Proteobacteria: alpha and beta subclasses. Springer, New York, NY. doi:10.1007/0-387-30745-1_9.

[2] Nanda K, Taniguchi M, Ujike S, Ishihara N, Mori H, Ono H, Murooka Y. 2001. Characterization of acetic acid bacteria in traditional acetic acid fermentation of rice vinegar (komesu) and unpolished rice vinegar (kurosu) produced in Japan. Appl Environ Microbiol 67:986–990. doi:10.1128/AEM.67.2.986-990.2001. 11157275 PMC92679

[3] Tanasupawat S, Kommanee J, Yukphan P, Muramatsu Y, Nakagawa Y, Yamada Y. 2011. *Acetobacter farinalis* sp. nov., an acetic acid bacterium in the α-*Proteobacteria*. J Gen Appl Microbiol 57:159–167. doi:10.2323/jgam.57.159. 21817828

[4] Pitiwittayakul N, Theeragool G, Yukphan P, Chaipitakchonlatarn W, Malimas T, Muramatsu Y, Tanasupawat S, Nakagawa Y, Yamada Y. 2016. *Acetobacter suratthanensis* sp. nov., an acetic acid bacterium isolated in Thailand. Ann Microbiol 66:1157–1166. doi:10.1007/s13213-016-1200-z.

[5] Pitiwittayakul N, Yukphan P, Chaipitakchonlatarn W, Yamada Y, Theeragool G. 2015. *Acetobacter thailandicus* sp. nov., for a strain isolated in Thailand. Ann Microbiol 65:1855–1863. doi:10.1007/s13213-014-1024-7.

[6] Lane DJ. 1991. 16S/23S rRNA sequencing, p 115–147. *In* Stackebrandt E, Goodfellow M (ed), Nucleic acids techniques in bacterial systematics. John Wiley & Sons, Chichester, United Kingdom.

[7] Bankevich A, Nurk S, Antipov D, Gurevich AA, Dvorkin M, Kulikov AS, Lesin VM, Nikolenko SI, Pham S, Prjibelski AD, Pyshkin AV, Sirotkin AV, Vyahhi N, Tesler G, Alekseyev MA, Pevzner PA. 2012. SPAdes: a new genome assembly algorithm and its applications to single-cell sequencing. J Comput Biol 19:455–477. doi:10.1089/cmb.2012.0021. 22506599 PMC3342519

[8] Tatusova T, DiCuccio M, Badretdin A, Chetvernin V, Nawrocki EP, Zaslavsky L, Lomsadze A, Pruitt KD, Borodovsky M, Ostell J. 2016. NCBI Prokaryotic Genome Annotation Pipeline. Nucleic Acids Res 44:6614–6624. doi:10.1093/nar/gkw569. 27342282 PMC5001611

[9] Zhang H, Yohe T, Huang L, Entwistle S, Wu P, Yang Z, Busk PK, Xu Y, Yin Y. 2018. dbCAN2: a meta server for automated carbohydrate-active enzyme annotation. Nucleic Acids Res 46:W95–W101. doi:10.1093/nar/gky418. 29771380 PMC6031026

[10] Johnson LS, Eddy SR, Portugaly E. 2010. Hidden Markov model speed heuristic and iterative HMM search procedure. BMC Bioinformatics 11:431. doi:10.1186/1471-2105-11-431. 20718988 PMC2931519

[11] Arkin AP, Cottingham RW, Henry CS, Harris NL, Stevens RL, Maslov S, Dehal P, Ware D, Perez F, Canon S, Sneddon MW, Henderson ML, Riehl WJ, Murphy-Olson D, Chan SY, Kamimura RT, Kumari S, Drake MM, Brettin TS, Glass EM, Chivian D, Gunter D, Weston DJ, Allen BH, Baumohl J, Best AA, Bowen B, Brenner SE, Bun CC, Chandonia JM, Chia JM, Colasanti R, Conrad N, Davis JJ, Davison BH, DeJongh M, Devoid S, Dietrich E, Dubchak I, Edirisinghe JN, Fang G, Faria JP, Frybarger PM, Gerlach W, Gerstein M, Greiner A, Gurtowski J, Haun HL, He F, Jain R, et al. 2018. KBase: the United States Department of Energy Systems Biology Knowledgebase. Nat Biotechnol 36:566–569. doi:10.1038/nbt.4163. 29979655 PMC6870991

